# The Body as an Object of Stigmatization in Cultures of Guilt and Shame: A Polish–Vietnamese Comparison

**DOI:** 10.3390/ijerph16162814

**Published:** 2019-08-07

**Authors:** Małgorzata Lipowska, Ha Truong Thi Khanh, Mariusz Lipowski, Joanna Różycka-Tran, Mariola Bidzan, Thu Tran Ha

**Affiliations:** 1 Institute of Psychology, University of Gdansk, 80-309 Gdansk, Poland; 2 Faculty of Psychology, University of Social Sciences and Humanities, Hanoi 336, Vietnam; 3 Department of Health Psychology, Gdansk University of Physical Education and Sport, 80-336 Gdansk, Poland

**Keywords:** body stigma, body image, cross-cultural psychology, gender differences

## Abstract

The aim of this paper is to examine cross-cultural differences in body stigmatization between the individualistic Christian culture of guilt (Poland) and the collectivistic Buddhist/Confucian culture of honor and shame (Vietnam). The study included 1290 university students from Poland (*n* = 586) and Vietnam (*n* = 704). Subjects filled in the body esteem scale and the perceived stigmatization questionnaire, and body measurements were collected to calculate anthropometric indices. Participants from Vietnam were less satisfied with their appearance than their Polish peers. Men in both countries assessed themselves more favorably. No anthropometric index predicted body esteem in Vietnamese women, while only indices related to fat levels were predictors in Polish women. Men with a V-shaped body assessed themselves as stronger and as having a better physical condition. A possible explanation of the observed cross-cultural differences is that interdependent self-construal makes young adults in collectivistic societies more susceptible to criticism, and the Confucian values of modesty and shame lead to them not perceiving their bodies as sexual objects. The Christian sense of guilt does not influence the perception of sexuality. Absence of friendly behavior mediated the relation between anthropometric indices and body esteem in both cultures.

## 1. Introduction

“Body Self” is the first component of the “Self” which is developed in the course of ontogenesis [1]. One’s relationship with one’s body starts to be defined as early as during early childhood, initially due to parental influences. Parents/caregivers determine their children’s relationship with physical attractiveness, both indirectly and directly, and influence its role in their child’s social life [2]. The creation of beauty ideals takes place during the process of socialization—parents/caregivers impart to their child patterns of beauty specific to their culture [3]. Moreover, parents/caregivers directly express their approval or disapproval of the appearance of other people, media celebrities, or their own children [4]. The role of this process is significant because unaccepted body appearance is one of the main reasons of stigmatization [5].

Self-perception of one’s body is not only associated with one’s sense of aesthetics but also represents one of the key elements of body image [6]. Age and gender are the two factors which most significantly affect the way the body is felt and perceived [7,8,9,10]; however, we postulate that culture is another such factor.

### 1.1. Age and Gender as Determinants of Body Perception

General observations on the course of development suggest that despite children being generally content with their appearance [11], some children would like to change some elements of their looks [3,12]. In adolescence and early adulthood, body image declines significantly, mostly as a result of feedback from one’s environment [2,10,13], it increases slightly in mid-adulthood [14], and at the threshold of old age, it rapidly declines as a result of being confronted with visible signs of aging, as well as stereotypes about old age [15,16].

However, it seems that the most significant difference observed is in the importance that men and women assign to perceived physical attractiveness [17]. Despite the fact that everyone, regardless of gender, pays attention to their bodies, men and women assign different levels of importance to the qualities of beauty and health. This gender difference is primarily associated with different attitudes towards one’s own body: Women are more likely to treat it as an object, and men are more likely to treat it as a process [18]. Considering the body as an object is associated with treating it as a set of independent, static elements (face, hands, figure, etc.), each of which is subject to independent assessment. Treating the body as a process emphasizes its functioning—the body is considered as a complete, well-functioning whole. Such different ways of treating one’s body are very visible during adolescence and are directly associated with gender stereotypes. As a consequence, the Body Self, and especially the body image of girls and women, is a much more important part of the “Self”, and it directly and significantly impacts overall levels of self-esteem [19,20]. This pattern applies to the attractiveness of men to a much lesser degree. Treating one’s body as a process results in less criticism with regard to appearance, which is of peripheral importance—one is satisfied with their body if it is capable enough. Many empirical studies confirm this and emphasize that the main indicator of a man’s attractiveness is his musculature, which is considered an indicator of the fitness and strength of his body [21]. Thus, well-defined muscles are assessed positively, but not overvalued [17,22].

### 1.2. Cultural Criteria of Body Image

The growing trend of dissatisfaction with appearance in individuals regardless of gender and age is being emphasized more frequently in the literature [23]. The beauty standards to which both men and women compare themselves are embedded in a society and its culture. The Tripartite Influence Model [24] and its many empirical confirmations [23,25,26,27]. indicate the existence of both direct and indirect sources of body dissatisfaction. Peers, parents, and the media directly influence one’s attitude towards one’s own body, while comparing appearances and internalization of society’s ideal body type have an indirect impact.

Figure and face are believed to be the two main indicators of physical attractiveness [28,29], but research clearly suggests that figure is the main criterion of physical attractiveness. When assessing their figure, women concentrate on their body mass and the shape of their body [8,22,30], while men concentrate on musculature [21,31,32].

While a muscular figure is a clear indicator of the attractiveness of a man’s body, research comparing women’s preferences with men’s beliefs about women’s preferences clearly indicate that men significantly overestimate the value that women ascribe to musculature [33]. Pursuing the ideal muscular figure may take on a pathological character, referred to as the *drive for muscularity* [34]—understood as a drive to increase muscularity by resorting to practices that are detrimental to one’s health, such as the use of anabolic steroids and strenuous physical exercise, ignoring the associated psychological and physical costs. As a consequence, problems such as depression, eating disorders, or addiction to physical exercise (*obligatory exercise*) can occur [35,36]. Body mass is said to be the main indicator of attractiveness in women, and it is also important for men [37,38]. While women generally want to lose weight, men often have two goals: wanting to lose excessive fat, and, more frequently, to gain muscle, which necessitates increasing one’s body weight [27].

Another predictor of perceived body image (in addition to age and sex) seems to be culture. Culture shapes the context in which body image is formed, and hence, it is a critical component to consider when understanding how body image changes [39]. Many studies identify the culturally-determined factors that contribute to body image, such as the media, peer influence, and family socialization [40,41,42,43]. For example, culture influences both the type and the content of the media to which people are exposed. Culture can also influence the type of people one bonds with and how parents raise their children.

The most pronounced difference in attitudes towards the body may be observed in the contrast between cultures of guilt and cultures of shame. The differences between these two types of culture lie in the values of their worldviews and religious systems, especially in their attitudes towards sex and nudity. In Christian cultures, the body is corrupted with original sin and needs to be cleansed through the act of baptism, after which the purity of body and soul needs to be maintained—any contamination induces a sense of guilt; some studies confirm that Christianity increases the sense of guilt [44]. By contrast, in Confucian or Buddhist cultures, the body is considered a part of human nature and a reflection of one’s soul (a damaged body indicates spiritual frailty); bodily appearance is key in social comparisons and indicates belonging to a given level in the hierarchical society (an unkempt appearance invokes shame). In collective cultures, there is also the phenomenon of “saving face”—“face” is defined both as the self-presented to others and as the self-perceived by others, mirroring the duality of self [45].

Research conducted in 37 countries [46] showed that in collectivist cultures (e.g., South America, Greece), a sense of shame was experienced more often than guilt, while in individualist cultures (e.g., the USA, Sweden), guilt was the dominating emotion. One can therefore distinguish between “shame societies”, where an individual is subordinate to society and avoids bad deeds due to fear of punishment, and “guilt societies”, where the autonomous individual avoids bad deeds out of respect towards norms they recognize.

Bierbrauer [44] referred to shame as a defensive response to the criticism of others, which stems from one’s fears of rejection and retraction of social support, whereas guilt is treated as self-criticism arising from failures to meet internalized standards. Wong and Tsai [47] suggested that shame involves being evaluated negatively by either real or imagined others (i.e., being focused on others and thus possessing an outward orientation), whereas guilt involves being evaluated by oneself negatively (i.e., being focused on oneself and thus possessing an inward orientation).

Cross-cultural studies confirmed that individualistic and collectivistic cultures use guilt and shame differently for social control. For instance, if one assumes that shame is a threat to the self, members of collectivistic societies may be expected to experience greater shame reactions than those of individualistic societies because the boundaries between the self and others, as well as those between public and private selves [48], are less well-defined. In other words, in collectivistic cultures, breaking group norms induces a sense of shame and guilt, whereas in individualistic cultures, breaking one’s own (internalized) moral norms induces shame and guilt.

### 1.3. The Body as an Object of Stigmatization in Cultures of Guilt and Shame

The inability to meet social demands for a perfect appearance can lead to decreased quality of life [49,50], is a correlate of disorders of a psychological/mental character [51,52], and is also the source of a sense of stigmatization and its associated social isolation [53,54,55,56,57]. Having a healthy body image is an important part of having a good life. For example, many studies have shown that a negative body image is related to destructive behaviors [6]. Other studies showed that a positive body image is related to better outcomes, such as happiness and life satisfaction [27].

Paradoxically, the highest level of criticism concerning one’s own body occurs during youth [13], often referred to as the so-called “social audience effect” because young people feel continuously assessed by others. Even minor deviations from the accepted canons of beauty or visible defects (scars, marks, etc.) become the basis of a sense of stigmatization. Stigma is defined as a social attribute, a mark, a feature that deeply discredits the individual who is perceived as defective. Sources of stigma can be bodily defects, lack of a strong will, or character flaws as well as tribal stigmas of race, religion, or nationality [58,59]. There are different ways in which appearance can be stigmatizing. Even though both obesity and facial imperfections or dermatological conditions do not fit into today’s canon of beauty, they are perceived significantly differently. Obesity can be considered “fixable” through exercise, diet, or drugs and thus to reflect on one’s willpower, while, by contrast, facial features and skin defects are understood to be often genetically determined and therefore beyond the control of the individual [60]. Obesity is therefore also stigmatized because of the implicit defects of character, such as, for example, intemperance in eating [61]. In the eyes of their peers, obese teenagers are not only externally unattractive but also lack the ability to maintain interpersonal relationships [62], and “fat” children are often referred to by their peers as “lazy” or “sloppy” [63]. It can be therefore concluded that obesity is stigmatized twofold.

Individuals from collectivist cultures [64] seem to be particularly prone to the influence of their social environment (the “social audience effect”) because of interdependent self-construal [65]. At the individual level, individualistic cultures shape the orientation of the self towards others (i.e., dependent versus independent), the relative importance of personal versus group goals, and the extent to which behavior is assumed to result from dispositional versus situational determinants [66]. An interiorized culture can shape not only perceptions of the self and others, but also emotional experiences [46]. Shame and guilt are distinctive self-conscious emotions that are inextricably linked to the relationship between self and others [67]. As shame and guilt are self-conscious emotions, evidence of differences between shame and guilt gathered from participants whose cultural orientation is individualist may not apply to individuals whose cultural orientation is collectivist. Thus, collectivist cultures are especially prone to stigma, which is associated with particular consequences, especially among teenagers [59].

### 1.4. The Current Study: A Polish-Vietnamese Comparison

The aim of this research was to verify whether there are different approaches to the body as an object of stigmatization in different cultural contexts: namely, the Christian culture of guilt and the Confucian culture of honor and sense of shame. The following research hypotheses were formulated on the basis of the theoretical premises:

**Hypotheses 1** **(H1).** *Culture and gender are moderators of body esteem and perceived stigmatization:*

- Young adults from the culture of shame (Vietnam) are less satisfied with their looks and experience higher levels of stigma in comparison to their peers from the culture of guilt (Poland); and
- Women are less satisfied with their bodies and experience higher levels of stigma than men.

**Hypotheses 2** **(H2).** *Objective body dimensions and anthropometric indices are predictors of body esteem and perceived stigmatization irrespective of the culture and gender of participants.*

**Hypotheses 3** **(H3).** *Perceived stigmatization is an important predictor of body esteem; the strongest dependence occurs in women from the culture of shame, while the weakest in men from the culture of guilt.*

### 1.5. Participants

Data were collected from *N* = 1290 participants, aged 19 to 25, in two countries: Poland and Vietnam. Polish students came from the University of Gdansk and the Gdansk University of Physical Education and Sport in Poland (*n* = 586): 437 women of mean age *M* = 21.22 (*SD* = 1.68) and 149 men of mean age *M* = 21.77 (*SD* = 1.69). Vietnamese students came from the University of Social Sciences and Humanities in Hanoi in Vietnam (*n* = 704): 461 women of mean age *M* = 20.43 (*SD* = 1.09) and 246 men of mean age *M* = 20.36 (*SD* = 1.11).

### 1.6. Procedure

Recruitment was performed using nonprobability sampling—age and education were the main inclusion criteria. The study was conducted after class in universities. Participants filled out questionnaires, and anthropometric measurements were taken by the research team. Study procedures took less than 45 min.

The protocol of this study was approved by the Ethics Board for Research Projects at the Institute of Psychology, University of Gdansk (decision no. 9/2017). According to the local law of different universities, no written permission from participants was required, as data were collected and analyzed anonymously. Participants were assured that their data would remain anonymous and confidential. The work described was carried out in accordance with the Code of Ethics of the World Medical Association (Declaration of Helsinki) for experiments involving humans using data collection.

## 2. Methods

The subjects’ attitude to their bodies was determined using the Body Esteem Scale [68] and sense of stigmatization was assessed using the Perceived Stigmatization Questionnaire [69]. Additionally, we used objective information from body measurements: weight, height, and sizes of individual body parts. This information allowed us to calculate anthropometric indices for all participants, such as body mass index (BMI), index of central obesity (ICO), as well as specific indicators: waist-to-hip ratio (WHR) and breast size for women, and shoulder-to-hip ratio (SHR) and waist-to-chest ratio (WCR) for men.

The Body Esteem Scale (BES) by Franzoi and Shields [68], adapted to Polish by Lipowska and Lipowski [70], is suitable for determining a respondent’s attitude to their body. The scale consists of 35 items grouped into three subscales, which are different for men and women. The *Sexual* Attractiveness, Weight Concern, and Physical Condition subscales are specific to women, whereas the Physical Attractiveness, Upper Body Strength, and Physical Condition are specific to men. Each BES statement is scored using a five-item Likert-type scale, where 1 corresponds to have strong negative feelings, 5 to have strong positive feelings, and 3 represents a neutral midpoint. The Sexual Attractiveness (women) and Physical Attractiveness (men) subscales involve assessment of those body parts which would be described as beautiful in a woman and as handsome in a man. Importantly, they refer to aspects that cannot be modified with physical exercise (e.g., self-satisfaction with one’s lips, breasts, feet). In turn, the Weight Concern subscale (women) involves assessment of body parts whose appearance can be modified by physical exercise or diet (waist, buttocks). The Upper Body Strength subscale (men) involves assessment of body parts (e.g., chest and arms) whose appearance and strength make a man appear strong and active. The Physical Condition subscale concerns the endurance, strength, and agility of one’s body. The body esteem scale is a widely used measure with acceptable reliability and validity, with Cronbach’s alpha varying between α = 0.81 and α = 0.86 for subscales.

The BES was adapted to Polish conditions by Lipowska and Lipowski [70]; the empirical scores for each subscale can be compared to the age-specific reference values determined in a group of more than 4000 subjects from Poland.

The Perceived Stigmatization Questionnaire (PSQ) [69] is used to assess the sense of stigma. Its reliability is confirmed by high values of Cronbach’s alpha = 0.93. In order to develop a Polish version of the PSQ, with the author’s consent, the questionnaire was translated into Polish independently by an interpreter and a psychologist. After selecting the best Polish version, it was backtranslated into English by a native speaker. Then, the quality of translation was assessed by comparing the backtranslation with the original questionnaire. The questionnaire is composed of 21 items which form 3 subscales: Absence of Friendly Behavior, Confused/Staring Behavior, Hostile Behavior, and Total Score, measuring the overall subjective sense of stigma. Participants have to assess how often people behave in certain ways around them on a 5-point Likert type scale, where 1 indicates never, 5 indicates always, and 3 indicates sometimes. The following satisfactory reliability/validity indices for given subscales were obtained in the presented study: Absence of Friendly Behavior α = 0.72, Confused/Staring Behavior α = 0.76, and Hostile Behavior α = 0.85.

As well as psychological tests, the following measurements were taken: participants’ height and body mass, as well as other body measurements, which allowed for the calculation of anthropometric indices. Indices related to body mass (body mass index and index of central obesity) were calculated for all participants, and indices related to body shape were calculated separately for men and women: waist-to-hip ratio and breast size for women, and shoulder-to-hip ratio and waist-to-chest ratio for men.

*Body mass index* (BMI), the ratio of body mass to height (body mass (kg)/height (m)²), is the most popular and widespread index used to classify a person as underweight, overweight, or obese. BMI is, however, a simple, and rough measure, as it does not take body composition into account. This is why it is inaccurate when assessing, inter alia, athletes, children, and pregnant women [71].

*Index of central obesity* (ICO) is the ratio of waist circumference to height, which allows the estimation of the proportion of visceral fat to total body fat. ICO may be applicable to children, and it is more accurate in assessing one’s health than BMI because the measurement concerns the abdominal area, which is the spot where most fat tissue is accumulated [72].

*Waist-to-hip ratio* (WHR) is the ratio of waist to hip circumference. It usually falls between 0.6 and 1.0, and the reference values are different for men and women due to sex differences in body fat distribution, resulting in different body shapes. The lower the WHR, the closer the body shape is to an hourglass figure—a feature which is socially desirable in women, but not men [73,74,75].

*Breast size* is obviously an index of body proportion which only concerns women. Most research, including cross-cultural studies [76,77], indicates that men emphasize the importance of breast size when assessing the overall attractiveness of a woman, and they consider large, or at least average-sized, breasts to be most attractive [78,79,80].

*Shoulder-to-hip ratio* (SHR) is concerned with upper body proportions. The upper body is usually larger in men, and so, just like WHR, SHR is an index associated with sexual dimorphism. Men are characterized by higher SHR than women; moreover, SHR correlates with upper body strength and greater handgrip strength [81,82].

*Waist-to-chest ratio* (WCR) is an index concerning the typically male body shape. A low WCR indicates a V-shaped body. Many studies suggest that these proportions are more important for men’s perceived attractiveness than their fat levels [83].

WCR and SHR are often used interchangeably in research, even though waist-to-chest may be more related to musculature, whereas shoulder-to-hip ratio is more related to one’s posture as determined by the width of the shoulder girdle.

## 3. Results

### 3.1. Anthropometric differences

Cross-cultural and gender differences in terms of anthropometric variables (body circumferences and the calculated indices) were analyzed first (Table 1).

Results suggest that Polish men and women are “bigger” in comparison to their Vietnamese peers—i.e., their body height and weight, as well as all measured circumferences, were bigger. Polish women also had bigger breasts and lower waist-to-hip ratio (WHR) than Vietnamese women, while Polish men had higher shoulder-to-hip ratio (SHR). Interestingly, the index of central obesity (ICO) was similar in the two countries for both men and women in this sample. A small difference between Polish and Vietnamese men was observed also in terms of waist-to-chest ratio (WCR).

### 3.2. Cross-Cultural Differences in Assessing one’s Body and Sense of Stigma

Attitudes toward one’s body seem to differ between Poland and Vietnam for both men and women (see Table 2). The greatest differences were observed for sexual/physical attractiveness—the lowest assessments were made by Vietnamese men and the highest by Polish women (all post-hoc differences: *p* < 0.001). Differences between countries, but not between genders, were also observed0 in weight concern/upper body strength. Both Polish women and men assessed themselves higher than their peers from Vietnam. In turn, gender differences were observed in terms of physical condition: men, regardless of country of origin, assessed their physical fitness higher. This score confirms to a large extent the first hypothesis—that young adults in the culture of shame (Vietnam) are less satisfied with their looks in comparison to their peers from the culture of guilt (Poland); moreover, in both countries, men assess themselves more favorably than women.

Results concerning the sense of stigma (see Table 2) indicate that absence of friendly behavior is reported more frequently by female students from Vietnam than from Poland. Differences were not observed either between men from the two countries or between men and women. The confused/staring behavior scores of men from Vietnam stand out—their perceptions of such behaviors are statistically higher (post hoc: *p* < 0.001) than in all other groups (no other group differences observed). The highest number of differences (both cross-cultural and gender) was observed for hostile behavior: Vietnamese men had the highest and Polish women had the lowest perceptions of such behavior. This result is in line with Hypothesis 1, which assumed that perceived stigma is influenced by both the gender and culture of origin of young adults.

### 3.3. Anthropometric Predictors of Satisfaction with one’s Body

**Figure 1:**
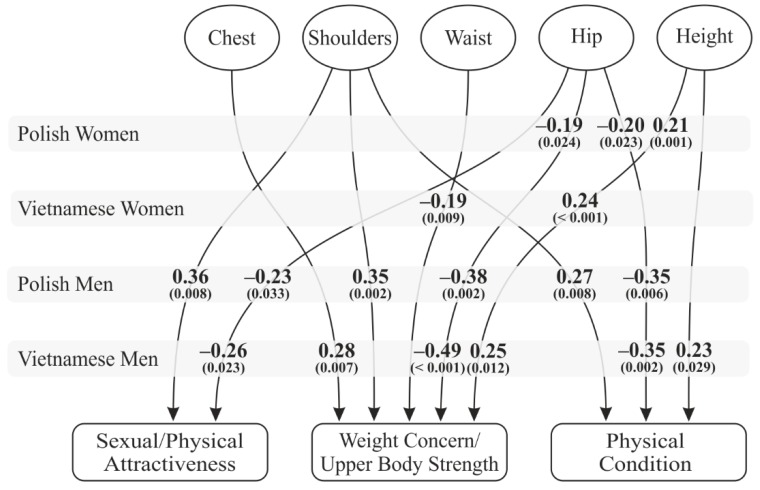
Body measurements as predictors of body esteem.

Seven body measurements were taken for women. Of these, bust, under-bust, and neck circumference, as well as body weight, turned out to be insignificant as predictors of satisfaction with one’s body. The dimensions which most influenced women’s assessment of weight concern and physical condition were the circumferences of waist and hips, as well as height (Figure 1). None of the measurements predicted the women’s satisfaction with their sexual attractiveness.

Significantly more predictors of body assessment were found for men—mainly shoulder and hip circumference. In both cultures, wide hips predicted poorer assessments of one’s body on all dimensions of the BES scale. However, shoulder circumference predicted body satisfaction in Polish men only. Neck circumference, waist circumference, and body weight all failed to predict body satisfaction in men.

**Figure 2:**
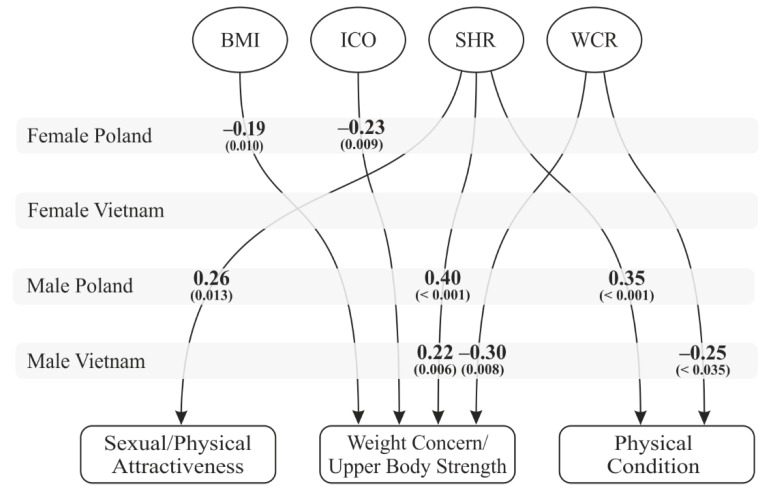
Anthropometric indicators as predictors of body esteem.

Anthropometric indices were calculated based on body measurements. With regard to these indices, the differences observed were mostly cross-cultural (Figure 2). None of the indices predicted any aspect of satisfaction with one’s own body in Vietnamese women. The main predictors of satisfaction with one’s body for Polish women were those associated with levels of body fat—which unsurprisingly predicted weight concern.

Cross-cultural differences were also observed in men. The only predictor found to be significant for Polish men was the SHR index, which influenced all aspects of satisfaction with their bodies. For Vietnamese men, the shoulder-to-hip ratio was important for the assessment of upper body strength, while the WCR index predicted assessments of upper body strength and physical condition.

The above suggests that the part of Hypothesis 2 regarding body esteem was mostly confirmed for men. Contrary to our assumptions, “feminine” body parts, such as bust, waist or hips, did not predict women’s body esteem.

### 3.4. Anthropometric Indices and Sense of Stigma

A surprising result was observed in that objective body measurements had no influence on women’s sense of stigma. In the case of men, body mass (β = 0.66, *p* < 0.001), shoulder circumference (β = −0.21, *p* = 0.024), and chest circumference (β = −0.31, *p* = 0.005) predicted confused/staring behavior, but only in Vietnamese men, *F*(7, 174) = 4.25, *p* < 0.001. Since significant results were observed for Vietnamese men, the second hypothesis needs to be rejected, and it must be concluded that body measurements did not predict perceived stigmatization independent of the culture and gender of participants.

### 3.5. Cultural Determinants of the Relationship Between Perceived Stigma and Attitude Towards one’s Own Body

**Figure 3:**
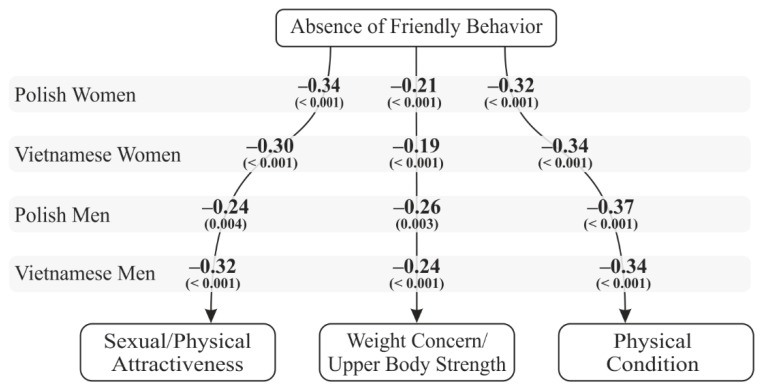
Absence of friendly behavior as a predictor of body esteem.

Further statistical analyses were done to verify whether sense of stigma is a significant predictor of body esteem. Bootstrapping mediation effects analysis did not show any of the dimensions of perceived stigmatization to mediate the relationship between body measurements and body esteem. Of the three predictors, only absence of friendly behavior was important for all indices of body assessment (Figure 3)—the bigger the absence of friendly behavior, the lower the assessment on the BES subscales.

Lack of friendly behaviors (i.e., compliments) and a lack of experiencing criticism turned out to predict body satisfaction independently of the gender and culture of participants. Thus, Hypothesis 3, which assumed that perceived stigmatization is a significant predictor of body esteem and that there are cross-cultural and gender differences in this prediction, must be rejected.

## 4. Discussion

The results are discussed in the context of culture as another determinant of body satisfaction.

*Anthropometric variables.* As expected, due to differences in ethnicity, the Polish participants turned out to be “bigger” than the Vietnamese participants, i.e., all their anthropometric measurements (circumferences, height, body mass) had higher values. Thus, the objective data collected indicate that measurements were taken correctly and can be treated as reliable predictors of subjective assessments of one’s body or sense of stigma.

*Gender and body esteem.* The results only partially confirmed the assumption that women from both cultures would be less satisfied with their bodies than men, which has been suggested by many previous results [13,68]. Interestingly, this difference was especially pronounced for body parts whose appearance cannot be modified with physical exercise or diet—i.e., those which are “given” or “inherited”. Body mass and body strength can be modified through an individual’s own efforts, and both women and men were generally satisfied with their mass and strength. Most men were satisfied with their fitness, which is in line with the assumption that men treat their bodies as an integral whole whose efficient functioning is of primary importance [18]. Only young individuals were included in the study, so it can be assumed that they do not complain about decreased functioning due to age. This makes the gender difference even more interesting, and it seems worthwhile to analyze the relationships between objective body measurements and body esteem.

*Anthropometric indices and body satisfaction.* Here, the results are very surprising. Among over 700 young women, only minimal relationships were found between objective body measurements and satisfaction with one’s body. Bust has previously been frequently mentioned as the sine qua non of womanhood [76], and here, it played no role at all. Waist and hip circumferences, associated with the so-called pear-shaped figure, were associated with satisfaction with body mass and physical fitness. No relationships between anthropometric indices and body esteem were found. It seems that it is not what one actually looks like that matters, but rather how one feels about one’s looks. It appears that young Vietnamese women’s assessments of their bodies are not influenced by their actual body shape, and body shape only has a small influence on women in Poland, for whom BMI was a significant predictor. This makes it seem even more worthwhile to examine the role of culture in detail.

Hip circumference was associated with lower body esteem in men—probably because bigger hip circumference is related to more a feminine body shape. Here, significant cross-cultural differences were observed for respective indices, but the general observation can be made that men assess their body strength and fitness more positively when they have a V-shaped body.

*Cross-cultural differences in body assessment.* Culture turned out to be a very important factor differentiating attitudes towards bodies: Young adults from the culture of shame (Vietnam) were less satisfied with their looks than their peers from the culture of guilt (Poland). It is possible to look for an explanation for this in the concepts about the body specific to Poland and Vietnam—the body is separate from the soul in the culture of guilt, while body and soul are one entity in the Confucian culture of shame. This could explain the result that Vietnamese women are more likely to perceive their sexual attractiveness through spiritual features (which are reflected in one’s body). In the culture of guilt, the body is the key factor in perceived attractiveness (due to the “impurity of the body” from the concept of original sin), while in the culture of shame, attractiveness may be associated with social roles (a sexually attractive woman is a good caretaker of the house and her man), while a sexually attractive man is a warrior or a breadwinner. Performing given roles or fulfilling expectations and duties towards other members of a collectivist society (e.g., filial piety) may have a bigger influence on the perception of one’s own attractiveness (i.e., being useful for others in the group) than looks. Another explanation might lie in the Confucian features which function as definitions of beauty in Eastern culture: An attractive woman is modest, quiet, and submissive to her man, while an attractive man is a warrior but is also caring towards his woman. An Asian woman does not perceive her body as an individual sexual object, more as “a part of a collective body”, serving to fulfill culturally imposed social roles and expectations. Confucianism very strongly defines the context of social functioning of people, which everyone must adhere to, often at the expense of their individual needs.

The observed differences between participants from Poland and Vietnam shown in the relationship between body measurements, anthropometric indicators, and body esteem confirmed that it is valid to analyze these variables from a cross-cultural perspective. It is particularly striking in this context that while we did not identify any anthropometric index which predicted attitudes towards the body in Vietnamese women, the results of Polish men and women are in line with current theories of attractiveness [19,84]: For Polish women, the main predictors of body satisfaction were indices associated with levels of fat (BMI and ICO, which predicted weight concern), and in Polish men, the main predictor was the SHR index, which positively correlated with body esteem (while in Vietnamese men, WCR played a bigger role and was correlated negatively with body esteem). The results of the Vietnamese group indicate that it is necessary to take into account cultural factors when discussing people’s assessments of their own bodies, especially in the context of the sexual attractiveness of women and physical attractiveness of men.

*The body as an object of stigmatization in cultures of guilt and shame.* The results revealed interesting patterns: Objective anthropometric indices were not significant predictors of perceived stigmatization in both cultures. However, regardless of the anthropometric indices, Vietnamese men and women scored higher on all dimensions of perceived stigmatization in comparison to Polish women and men. Vietnamese male and female students reported higher levels of absence of friendly behaviors, confused/staring behavior, and hostile behavior. This might be explained by the fact that individuals from collectivist cultures [64] seem to be particularly prone to the influence of their social environment (the “social audience effect”) because of interdependent self-construal [65].

Interestingly, of the three dimensions of perceived stigmatization, only absence of friendly behaviors (i.e., compliments) was a significant mediator of the relationship between objective anthropometric indices and subjective perceptions of one’s body in both cultures. Neither confused/staring behavior nor hostile behavior (which were more often reported in the collectivist culture of shame) influenced the perceived stigmatization associated with objective body measurements. This means that compliments (and not criticism) influence perceived stigmatization, regardless of cultural context (shame or guilt). This is an important result, which contributes to the existing body of literature which has previously mainly emphasized the influence of criticism on stigmatization [5,55,85,86]. Thus, the need to stress the importance of complimenting an individual’s looks is a crucial implication of our study.

The main conclusion is that culture influences perceptions of one’s own body and sense of stigmatization: Young adults in collectivistic societies are more fragile to criticism concerning their bodies, likely because of interdependent self-construal, and do not perceive their bodies as sexual objects because of Confucian concepts of modesty and shame. On the other hand, the sense of guilt typical of Christian culture does not influence the perception of sexuality. However, in both cultures, compliments play an important role in body esteem.

### Limitations and Future Research

This study was conducted on groups of students, which is not a sample representative of an entire society. However, it should be noted that this is the demographic in which the highest levels of self-criticism of one’s body are observed. Another limitation is lack of measure consisting religious beliefs and culture of origin/ethnicity of the university students. They might come from cultures determined by older religions, but with secularization, they might be agnostics and also people from different ethnicities among the sample. Further, we did not control any possible influence of the type of university and courses on the body image. For example, sports students might be highly associated with their bodies and ideal of muscularity in comparison with social science students. Sport university male and female students constitute a specific group who undertake continuous physical training and this way they might treat their bodies as a means of process [87]. It should be investigated in future studies.

As our study reveals no direct relationships between anthropometric indices and body esteem, some mediators should be investigated, e.g., positive experience about the appearance or any religious beliefs which hardly influence the perception and social behavior [88]. Our results suggest that it would be worthwhile to continue analyzing the relationship between body esteem and both negative and positive feedback about one’s appearance. It would be interesting to investigate the influence of such positive feedback on emotions and cognitive self-perception independently from objective indices. As collectivistic societies seem to be more susceptible to criticism, it seems valuable to discover the tool of positive reinforcements for teenagers. In the future, it would also be interesting to look at the cultural and maybe religion factors responsible for perceptions of sexual attractiveness, especially in Asian women. Our findings suggest that body esteem is less important for Asian students (in comparison with western students); however, we do not know if it is a case of cultural values [89] or just being a student. Another case is that sexual attractiveness should be indicated especially among different subcultures in Asia, also in the context of perceived weight stigma, eating behavior, and psychological or emotional distress. For example, Hong Kong and Taiwan are Westernized areas with a traditional Chinese culture; this may be different from mainland Chinese and the Vietnamese population collected in the submitted work [90,91].

Another point is that we only studied the perceived stigma. Future works should concentrate on understanding self-stigma among people with higher weight (or excess weight) [92], using culture adapted tolls, e.g., the weight self-stigma questionnaire or weight bias internalization scale, which have been developed and widely used in research studies, including Asian countries [93,94].

To make such an investigation more applicable, we should develop some psychological interventions preventing stigmatization, especially among teenagers. In conclusion, it seems worthwhile to further investigate cultural, psychological, and sex differences in body image.

## Figures and Tables

**Table 1:** Culture and gender dependent differences in anthropometric measurements.

Feature/Index	Polish Women				Vietnamese Women				Differences	
	*M*	Min	Max	*SD*	*M*	Min	Max	*SD*	*t*	*p*
Neck	32.77	28.00	42.00	2.17	31.51	28.00	42.00	2.08	7.97	***
Breast	89.13	68.00	114.00	7.30	82.57	66.00	120.00	5.59	14.21	***
Below Brest	76.58	57.00	100.00	6.42	72.98	59.00	100.00	5.29	7.99	***
Waist	71.97	58.00	98.00	7.26	67.76	55.00	109.00	6.38	8.03	***
Hip	93.05	68.00	120.00	7.85	85.99	60.00	120.00	6.81	12.83	***
Height	167.83	147.00	189.00	6.01	157.39	140.00	180.00	5.31	24.28	***
Body Mass	60.03	40.00	95.00	9.27	48.28	36.00	80.00	6.02	20.24	***
BMI	21.28	15.17	32.77	2.80	19.50	14.48	31.64	2.15	9.45	***
ICO	0.43	0.33	0.59	0.04	0.43	0.33	0.69	0.04	0.86	
WHR	0.77	0.53	0.97	0.06	0.79	0.63	1.08	0.07	3.38	***
Breast Size	1.17	1.00	1.56	0.07	1.13	1.01	1.36	0.05	8.03	***
	**Polish Men**				**Vietnamese Men**				**Differences**	
Neck	38.85	29.00	46.00	3.03	35.73	29.00	46.00	2.71	9.68	***
Shoulders	116.39	98.00	134.00	8.02	104.51	82.00	136.00	7.53	13.26	***
Chest	100.20	70.00	119.00	9.07	87.86	70.00	110.00	6.94	13.74	***
Waist	84.06	60.00	118.00	9.80	76.31	59.00	122.00	9.39	7.41	***
Hip	96.21	74.00	120.00	8.90	91.50	68.00	140.00	9.13	4.73	***
Height	181.28	150.00	202.00	7.60	168.90	140.00	187.00	7.66	15.48	***
Body Mass	78.06	40.00	110.00	12.57	59.94	36.00	116.00	10.08	15.52	***
BMI	23.67	16.92	32.33	3.05	20.92	16.00	38.76	2.65	9.30	***
ICO	0.46	0.32	0.66	0.05	0.45	0.34	0.71	0.05	2.45	*
SHR	1.22	1.00	1.54	0.11	1.15	0.89	1.57	0.10	5.33	***
WCR	0.84	0.57	1.09	0.10	0.87	0.67	1.11	0.07	3.26	**

*Note.* BMI—body mass index, ICO—index of central obesity, WHR—waist-to-hip ratio, SHR—shoulder-to-hip ratio, WCR—waist-to-chest ratio, * *p* < 0.05, ** *p* < 0.01, *** *p* < 0.001.

**Table 2:** Cross-cultural and gender differences of body esteem and perceived stigmatization.

Features		PL ♀		VN ♀		PL ♂		VN ♂		Differences		
		*M*	*SD*	*M*	*SD*	*M*	*SD*	*M*	*SD*	Between:	*F*	*p*
**BES**	Sexual/Physical Attractiveness	48.01	7.14	43.38	6.24	41.56	6.38	39.09	6.85	♀ *vs.* ♂ VN *vs.* PL	160.96 112.85	*** ***
	Weight Concern/ Upper Body Strength	33.52	8.22	32.21	6.59	34.39	6.07	32.33	5.87	♀ *vs.* ♂ VN *vs.* PL	1.02 15.06	***
	Physical Condition	30.97	6.08	30.85	5.80	49.15	8.72	47.46	8.14	♀ *vs.* ♂ VN *vs.* PL	1754.04 2.30	***
**PSQ**	Absence of Friendly Behavior	2.29	0.42	2.42	0.46	2.36	0.52	2.43	0.49	♀ *vs.* ♂ VN *vs.* PL	1.62 18.86	***
	Confused/Staring Behavior	1.96	0.54	2.01	0.52	1.94	0.57	2.14	0.59	♀ *vs.* ♂ VN *vs.* PL	5.07 8.66	* **
	Hostile Behavior	1.51	0.52	1.81	0.62	1.65	0.65	1.96	0.68	♀ *vs.* ♂ VN *vs.* PL	15.08 80.91	*** ***

*Notes.* * *p* < 0.05, ** *p* < 0.01, *** *p* < 0.001.
